# Native Language Influence on Brass Instrument Performance: An Application of Generalized Additive Mixed Models (GAMMs) to Midsagittal Ultrasound Images of the Tongue

**DOI:** 10.3389/fpsyg.2019.02597

**Published:** 2019-11-27

**Authors:** Matthias Heyne, Donald Derrick, Jalal Al-Tamimi

**Affiliations:** ^1^Speech Laboratory, Department of Speech, Language & Hearing Sciences, College of Health & Rehabilitation Sciences: Sargent College, Boston University, Boston, MA, United States; ^2^New Zealand Institute of Language Brain and Behaviour, University of Canterbury, Christchurch, New Zealand; ^3^Speech and Language Sciences, Newcastle University, Newcastle upon Tyne, United Kingdom

**Keywords:** laboratory phonology, speech motor control, ultrasound imaging of the tongue, brass instrument performance, motor memory, acoustic to articulatory mapping, generalized additive mixed models (GAMMs), dispersion theory

## Abstract

This paper presents the findings of an ultrasound study of 10 New Zealand English and 10 Tongan-speaking trombone players, to determine whether there is an influence of native language speech production on trombone performance. Trombone players’ midsagittal tongue shapes were recorded while reading wordlists and during sustained note productions, and tongue surface contours traced. After normalizing to account for differences in vocal tract shape and ultrasound transducer orientation, we used generalized additive mixed models (GAMMs) to estimate average tongue surface shapes used by the players from the two language groups when producing notes at different pitches and intensities, and during the production of the monophthongs in their native languages. The average midsagittal tongue contours predicted by our models show a statistically robust difference at the back of the tongue distinguishing the two groups, where the New Zealand English players display an overall more retracted tongue position; however, tongue shape during playing does not directly map onto vowel tongue shapes as prescribed by the pedagogical literature. While the New Zealand English-speaking participants employed a playing tongue shape approximating schwa and the vowel used in the word ‘lot,’ the Tongan participants used a tongue shape loosely patterning with the back vowels /o/ and /u/. We argue that these findings represent evidence for native language influence on brass instrument performance; however, this influence seems to be secondary to more basic constraints of brass playing related to airflow requirements and acoustical considerations, with the vocal tract configurations observed across both groups satisfying these conditions in different ways. Our findings furthermore provide evidence for the functional independence of various sections of the tongue and indicate that speech production, itself an acquired motor skill, can influence another skilled behavior via motor memory of vocal tract gestures forming the basis of local optimization processes to arrive at a suitable tongue shape for sustained note production.

## Introduction

Brass instrument performance and speech production both require fine motor control of the vocal tract. Dalla Casa (1584/1970) made the connection centuries ago, using speech syllables in his method book for the Renaissance cornetto, a finger-hole trumpet, and, more recently, brass players have also suggested an influence of native language and culture on playing style (i.e., see Fitzgerald, 1946). Anecdotal accounts of language influence on brass playing exchanged within the brass playing community, for example, include speculation that players of some nationalities are ‘better’ than others at certain facets of brass playing or why learners may face specific challenges related to their language background (Heyne, 2016).

However, despite this pedagogical connection between brass instrument playing and speech, the connection between speech articulation and note production has been largely untested. Here we use ultrasound images of the tongue from ten New Zealand English and ten Tongan-speaking trombone players, to determine whether there is an influence of native language speech production on trombone performance. We investigated midsagittal tongue shape during note production by New Zealand English and Tongan trombone players, as well as the relationship between vowel and note tongue shapes within each language, and how the latter are affected by pitch and note intensity (loudness). The specific trombone pitches produced by participants in the study were Bb2, F3, Bb3, D4 and F4 (in ascending order, specified according to the US standard system where C1 refers to the lowest C on the piano) while the recorded intensities ranged from *piano* (soft) via *mezzopiano* and *mezzoforte* to *forte* (loud).

Following the earliest published account by Dalla Casa (1584/1970), countless brass players have continued to employ speech syllables in brass teaching, recommending the use of different consonants (/t/ versus /d/ for hard versus soft attacks) and, starting in the 19th century, vowel colors (/ɑ/ versus /i/ for low versus high range notes) to illustrate what students should do with their tongue to produce favorable sounds on brass instruments (cf. Heyne, 2016, section “2.4.1.2 Pedagogical writing on brass playing published in the last 50 years”). We have not come across any brass method books recommending the use of the ‘neutral’ vowel schwa, although it would seem to be an obvious candidate for achieving a maximally open (and uniform) vocal tract configuration as advocated by many influential teachers, perhaps most notably Arnold Jacobs, tuba player of the famous Chicago Symphony Orchestra (see Frederiksen, 2006; Loubriel, 2011). Most likely, the explanation is the lack of a consistent representation of schwa in standard orthography, and few highly accomplished brass players would have received formal training in linguistics or phonetics to raise such awareness. Of course, many of the world’s languages also do not have such a vowel quality.

Beginning in 1954, a small number of researchers started to empirically test the assumptions underlying the use of speech syllables in brass instrument pedagogy. Hall’s ground-breaking study (Hall, 1954) found that different players used unique individual positions of the tongue and jaw during trumpet performance, and that they tended to be consistent in using the same basic formation in all registers, indicating that no large modifications took place when changing registers. The author also traced midsagittal images of extreme vowels (“ah” [/ɑ/], “oo” [/u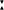/], and “ee” [/i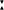/]) and reported that the most commonly used tongue shape during playing was “ah” but some players used the “oo” formation or intermediate formations falling between the extreme vowels. Subsequent work by Meidt (1967), Haynie (1969), Amstutz (1977), Frohrip (1972), and De Young (1975) largely confirmed Hall’s findings, while observing a wider range of playing conditions that included changes in loudness and note articulations/attacks (cf. Heyne and Derrick, 2016b); two of these studies, Frohrip (1972) and De Young (1975), observed trombone players exclusively. Notably, Hiigel (1967) asked his participants (players of all brass instruments) to ‘think’ prescribed syllables printed underneath the music while performing various notes and found no evidence “that thinking a syllable during performance will tend to simulate the tongue position resulting from the enunciation of that syllable” (p. 108). Rather, he found significant differences between tongue placement during playing and enunciation of the prescribed syllables and this was true even for the players who claimed to use those specific syllables while playing. Overall, there was a tendency for the “tongue arch” to be placed higher with the tongue tip “farther forward” when comparing playing to recitation (p. 107). Most studies, however, did not compare tongue shape during playing to speech production and the few that did used isolated vowel articulations which we now know are not representative of the patterns occurring in natural speech (Lindblom, 1963; Farnetani and Faber, 1992; DiCanio et al., 2015; Tsukanova et al., 2019).

Empirical research on vocal tract movements during brass playing stopped almost completely after the dangers of exposure to radiation from x-rays became apparent in the 1970s and until methods like ultrasound imaging and articulography became available (see Heyne and Derrick, 2016b). There exist two relatively recent Doctor of Musical Arts dissertations that investigated the influence of native language (Mounger, 2012) and dialect (Cox, 2014) on trombone performance more specifically; however, both studies only analyzed the acoustic signal produced during speech and instrument performance. Youngs (2018) presents a recent application of ultrasound tongue imaging to trumpet playing with a pedagogical focus which, however, involved comparisons of vowel and playing tongue shapes.

In comparison, speech production represents a well-researched field and it is both obvious and well-documented that speech differs across languages, dialects, and accents. Among the large number of possible speech sounds occurring in the world’s languages, vowel sounds have received the most attention, not only because they occur in every language but also because they are fairly easy to measure using both acoustic (Boersma and Weenink, 2014) and articulatory methods (Tiede, 2010; cf. Noiray et al., 2014; Tiede and Whalen, 2015). While some languages distinguish as few as three vowel sounds (Maddieson, 2013), other languages have up to 24 vowels (Maddieson, 1984; Vallée, 1994) and theoretical investigations suggest an effect of vowel inventory size on the general organization of vowel systems (de Boer, 2000).

More specifically, *Dispersion Theory* (Liljencrants and Lindblom, 1972; Lindblom, 1986) claims that speech sound organization is ruled by an “Adaptive Dispersion” of their elements, that follows a “Sufficient Perceptual Contrast” principle whereby acoustic vowel spaces are organized in a way that keeps them sufficiently distinct on the perceptual level. According to this theory, the phonetic values of vowel phonemes in small vowel systems should be allowed to vary more than in vowel systems with a more crowded vowel space. In addition, the *Quantal Theory of Speech* (Stevens, 1972; Stevens and Keyser, 2010) states that there are certain regions of stability in phonetic space, corresponding to the point vowels [i], [a], and [u]. Such vowels should be situated in approximately the same location across all languages, irrespective of vowel inventory size, and should display less intra-category variability than other vowels.

Both theories have received some empirical support (Al-Tamimi and Ferragne, 2005), which is unsurprising given they are informed by different investigative frameworks, namely speech perception as indexed by speech acoustics in the case of Dispersion Theory, and speech production represented by modeled vocal tract movements, in the case of the Quantal Theory of Speech. In addition, if [i], [a], and [u] inhabit regions of stability in phonetic space, then languages with larger vowel inventories must necessarily have regions of stability that separate vowels as clearly as in languages with smaller vowel inventories, which necessarily reduces variability of each vowel in larger vowel inventory systems. However, there are counter-examples for both Quantal and Dispersion Theory that relate to language variability (Bradlow, 1995) and the fact that not all three-vowel systems are maximally dispersed (Butcher, 1994), so a proper analysis must test both the range and variability of vowels and notes.

In addition, *Articulatory Phonology* (AP; Browman and Goldstein, 1986, 1992; Goldstein and Fowler, 2003) provides a theoretical framework whereby phonological units can be analyzed as constrictions occurring at various locations along the vocal tract. Six distinct ‘constricting devices’ (lips, tongue tip, tongue body, tongue root, velum, and larynx) form a combinatoric system of ‘gestures’ which minimally contrast at a single constriction location, and such gestures can overlap temporally, as modeled within the theory of *Task Dynamics* (Saltzman, 1986, 1995). Vowels are understood to differ mainly according to their constriction degree at locations involving the tongue (and lips) and as such are subject to the influence of preceding and following (consonant) articulations expressed by a ‘gestural score’ that indicates the organization of individual constricting movements and their patterns of coordination.

Although AP posits that speech should be regarded through a unitary structure that captures both physical (movement) and phonological properties, the underlying constriction actions are nonetheless potentially transferable across different vocal tract activities since they are described on the basis of goals rather than the resulting acoustic signal. Both in speech (phonology) and in brass instrument playing, gestures are geared toward the goal of producing behavioral outcomes that allow perceivers to distinguish between possible intended goals. Additionally, and similarly to speech, patterns of ‘coproduction’ (overlap of gestures) may occur during brass playing (a consonant-like gesture employed to start a note would overlap a vowel-like gesture during its steady-state) and could be governed by similar biomechanical properties. A possible mechanism for the transfer of vocal tract gestures across different vocal tract activities is provided by the concept of *motor memory*. Motor memory (alternatively *muscle memory*) is generally defined as “the persistence of the acquired capability for performance,” while the exact nature of the concept could refer to a “motor program, a reference of correctness, a schema, or an intrinsic coordination pattern” (Schmidt and Lee, 2011, pp. 461–462). Although it is of yet unknown where or how exactly such motor memory may be encoded and stored in the organs controlling human movement (see Tourville and Guenther, 2011, for suggestions regarding speech production), various researchers have suggested that the nervous system establishes muscular modules or “spatially fixed muscle synergies” (Ting et al., 2012) to reduce the excessive number of degrees of freedom observed during body motion (Bernstein, 1967; cf. Bizzi and Cheung, 2013). Furthermore, vocal tract movements seem to feature even greater muscle complexity than the rest of the human body (e.g., Sanders and Mu, 2013). It has been repeatedly demonstrated that speech production requires feedforward control (e.g., Neilson and Neilson, 1987; Perkell, 2012; Guenther, 2016) and operates in a multidimensional control space (Houde and Jordan, 1998; Tremblay et al., 2008; Gick and Derrick, 2009; Ghosh et al., 2010; Perkell, 2012), both of which are probably also true for brass instrument performance (see Bianco et al., 2010, for some evidence of the requirement of feedforward control when performing at maximum intensity on the trumpet).

Comparing the acoustic signal of brass instrument performance and vocalic speech production, one notices a similar pattern of steady states in sustained production, and dynamic changes in sound quality at the beginning and end of notes and vowels. There are also notable parallels in terms of how sound is generated during either activity. During brass playing, an outward-striking lip-reed mechanism – the player’s ‘embouchure’ – excites the air column within the instrument, producing a spectrum of standing waves which are controlled by the natural frequencies of the air column and which are emitted from the bell at varying volumes (Benade, 1976; Campbell and Greated, 1987).

The embouchure thus serves as the ‘source,’ comparable to the larynx during speech production, while the instrument bore serves as ‘filter.’ Unlike during speech production, however, the player has only limited means of altering the properties of this filter. On most brass instruments, the player can only alter the length of the ‘filter,’ which thus effectively serves merely as an amplifier. The much greater length of tubing compared to the human vocal tract also means that the possible resonating frequencies of the tube are very much determined by the overtone series except for very high registers where the peaks of the impedance spectrum become progressively smaller (cf. Hézard et al., 2014; see Wolfe, 2019 for an excellent non-technical description of brass instrument acoustics).

Nonetheless, the shape of the player’s vocal tract might influence the sound coming out of the instrument in limited ways, similar to the influence of subglottal resonances on speech production discovered only quite recently (Chi and Sonderegger, 2007; Lulich, 2010). While the pitch produced in the altissimo register of saxophones seems to be almost entirely determined by vocal tract resonances (Chen et al., 2008, 2012; Scavone et al., 2008), such resonances have a much smaller impact on brass instrument sound. Wolfe et al. (2010), observing the playing behavior of “an artificial trombone playing system,” found that “raising the tongue, or the tongue tip, increases the height of peaks in the vocal tract impedance, and so more effectively couples it to the instrument resonances” and the sound generating mechanism (p. 310). Crucially, this difference was observed without changing any other parameters, suggesting that the mechanism might provide players with a method of fine pitch adjustment.

A small number of studies have addressed the influence of vocal tract shaping on brass instrument sound in human subjects by “measuring the impedance spectrum of the vocal tract by injecting a known broadband acoustic current into the mouth” (Wolfe et al., 2015, p. 11); this requires notes to be sustained for roughly a second but it is then possible to directly determine vocal tract resonances during playing. Using this method, a team of researchers at the University of New South Wales in Australia measured vocal tract influence on trumpet (Chen et al., 2012) and trombone performance (Boutin et al., 2015). Both studies yielded similar results with impedance peaks in the vocal tract usually being smaller than those measured for the trumpet or trombone bore, although vocal tract resonances were less variable in trombone players. While this suggests that there is no systematic tuning of vocal tract resonances to influence instrument pitch (or possibly timbre), Chen et al. (2012) nevertheless speculate that raising the tongue, if not for vocal tract tuning, might facilitate high note playing by changing the magnitude or phase of vocal tract resonances (p. 727). In the specific case of the trombone (Boutin et al., 2015), the first vocal tract resonance consistently stayed within a narrow range of 200–375 Hz, leading the authors to conclude that those changes were mostly driven by changes in glottis opening (but see section “Other Constraints on Tongue Shape During Brass Instrument Performance” for conflicting findings on glottal aperture during brass instrument performance); the second vocal tract resonance, however, could “presumably be modified by varying the position and shape of the tongue, as is done in speech to vary the resonances of the tract” (p. 1200). Additionally, the authors noted a split across study participants by proficiency level: beginning trombone players more often produced second vocal tract resonances around 900 Hz while that number was around 650 Hz for advanced players. Interpretation of these results based on the first vowel formant in speech (F1, corresponding to the second vocal tract resonance peak as measured in this study) suggests the use of a lower tongue position by more proficient players. The same research group has also mentioned and, to a limited extent, investigated the possibility of vocal tract resonances influencing the timbre of wind instruments; although not determining or noticeably affecting the frequency of the fundamental of a played note, such a “filtering effect, though smaller for most wind instruments than for voice,” would admit the flow of acoustical energy into the instrument at some frequencies while inhibiting it at others (Wolfe et al., 2009, p. 7–8). The effect has been shown to determine the timbre of the didgeridoo (Wolfe et al., 2003) but it is much weaker on the trombone due to its higher impedance peaks and an additional formant introduced by the mouthpiece (cf. Wolfe et al., 2003, 2013).

It has also been suggested that vocal tract resonances could become dominant in the very high register of brass instruments. Based upon numerical simulations of simple and two-dimensional lip (embouchure) models, Fréour et al. (2015) propose a possible mechanism whereby changing the relative phase difference of oscillations within the oral cavity and instrument can lead to an optimum tuning of the system that maximizes acoustical feedback of oscillations within the instrument on the player’s lips, at the same time maximizing lip motion and hence the acoustic flow into the instrument.

In general, however, brass playing requires a larger amount of airflow (460 ml/s for a low note played on the trumpet at medium intensity; cf. Frederiksen, 2006, pp. 120–121; Kruger et al., 2006; Fréour et al., 2010 for information on other brass instruments) than speech production (around 150 ml/s during reading; Lewandowski et al., 2018) which may bias tongue position and affect the biomechanics of consonant-like tongue movement used to initiate notes. Students are usually taught to begin notes by releasing the tongue from a coronal place of articulation (although multiple articulations also make use of more retracted places of articulation so that attacks can occur in quick succession). In terms of a possible overlap of vocal tract movements during both activities, and hence the possibility of language influence on brass instrument performance, there are thus two possible areas of investigation: vowel production and its influence on steady states during brass playing, and the dynamics of consonant articulations on the way players begin and end notes on brass instruments.

The above-mentioned sparsity of empirical studies on vocal tract movements during brass instrument performance points to the difficulty of collecting such data (cf. Heyne and Derrick, 2016b). Ultrasound imaging of the tongue is a technique that has experienced increased use in the area of speech production research due to having no known side-effects (Epstein, 2005) and its comparably low cost (Gick, 2002) compared to more invasive technologies like real-time magnetic resonance imaging (MRI; e.g., Niebergall et al., 2013). Ultrasound imaging uses ultra-high frequency sound ranging from ∼3 to 16 MHz to penetrate soft tissues and calculate an image of their density by evaluating the echo returned when sound waves get reflected due to changes in tissue density; it was first applied to image the human tongue by Sonies et al. (1981). To produce ultrasound signals, ultrasound machines use piezoelectric crystals embedded in a transducer (or probe), which is held underneath the chin (submentally) when performing lingual ultrasound. Ultrasound waves “get absorbed by bone and reflect sharply off of air boundaries,” meaning that the technique does not image bone or air very well (Gick et al., 2013, p. 161); its second property, however, is very useful for imaging the shape of the tongue within the oral cavity as it provides good resolution of the tongue surface as long as there is continuous tissue for the sound waves to travel through.

**FIGURE 1:**
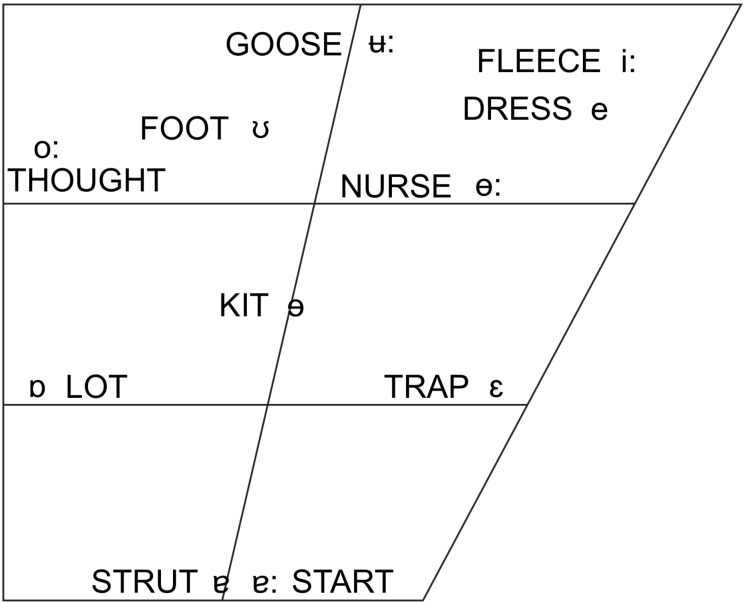
The monophthongs of New Zealand English. Note that this image is reprinted here in mirrored orientation from its source (Hay et al., 2008, p. 21) to match the orientation of the articulatory data presented in this paper.

On the basis of the considerations laid out above, we chose to conduct our study on brass players from two languages that differ significantly in the size and organization of their vowel systems. New Zealand English (NZE) is a Southern-hemisphere variety of English that features a phoneme inventory typical for English. Although many of its vowels are considerably shifted from the more well-known vowel systems of American and British English, it retains the same large number of monophthong vowel phonemes (Hay et al., 2008). Tongan, in contrast, is a typical Polynesian language with a small phoneme inventory that distinguishes only the five cardinal vowels /a, e, i, o, u/. See Table 1 and Figure 1 for additional detail on the phonological inventory of both languages; throughout this paper, we employ the lexical sets included in Figure 1 to refer to the vowel phonemes of NZE (cf. Wells, 1982). We also decided to focus on the trombone rather than including players of all brass instruments since differently sized mouthpieces might affect tongue shape due to varying air flow requirements and the potential for vocal tract influences on instrument sound at different resonating frequencies. Furthermore, the trombone provides an optimal choice in terms of investigating the influence of the dynamics of consonant articulations in speech on the way players begin and end notes (although this was not investigated in this study); in contrast to valved brass instruments such as the trumpet, a trombone player has to produce all articulations by momentarily interrupting the airflow into the mouthpiece (using the tongue and/or glottis) so that the researcher can be sure of the vocal tract contributions to such articulations.

**TABLE 1:** Tongan consonant **(left)** and vowel **(right)** inventories (from Garellek and White, 2015, 15).

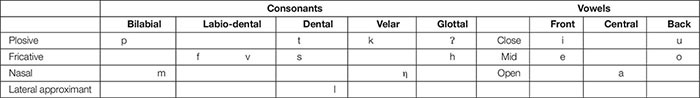

*Symbols appearing on the right sides of cells are voiced, symbols on the left are voiceless.*

In a previous analysis of a subset of this data (Heyne, 2016), we had used smoothing splines analysis of variance (SSANOVA; Gu, 2013b; package gss: Gu, 2013a) to calculate average tongue shapes for monophthong and sustained note productions in polar coordinates on the language group level and for each individual player; see Heyne and Derrick (2015c) and Mielke (2015) for discussions on why performing these calculations in Cartesian coordinates leads to errors that are most pronounced at the tongue tip and root. However, ultrasound data of speech production pose another serious issue: Analysis of tongue contour data has proven to be quite difficult, in part because appropriate techniques for recognizing accurate between-subject variation have historically been underdeveloped. SSANOVAs make assumptions about confidence intervals that are not statistically appropriate, so we decided to instead use generalized additive mixed-effects models (GAMMs) for our analyses presented in this paper.

Generalized Additive Mixed-effects Models (GAMMs) represent a statistical technique that deals with non-linear relationships between time-varying predictors and outcome variables (Hastie and Tibshirani, 1986; Wood, 2006, 2017). The technique has received attention within the phonetics community recently with major publications featuring the use of GAMMs to quantify the dynamics of formant trajectories over time from an acoustic point of view (Sóskuthy, 2017), or tongue position changes measured over time (Wieling et al., 2016; Wieling and Tiede, 2017). GAMMs work by applying a smoothing function (henceforth simply ‘smooth’) to a time-series that can be adjusted to the specific variables that may influence it. It is also possible to model random effects to take into account the inherent variability between, e.g., speakers and lexical items. Tongue contours obtained using Ultrasound Tongue Imaging are dynamic in nature as the full tongue contour is traced sequentially, and hence this can mimic the behavior of time-varying outcomes. Modeling tongue contours and change over-time is also possible by using a “tensor product interaction” (ti) between two different “time-series” (see Al-Tamimi, 2018).

Having established both the scope of the study, and the tools for analysis, we here outline a reasonable set of predictions for our hypotheses:

**Hypothesis 1:** A brass player’s native language will influence the vocal tract states they assume during performance on their instrument.
**Prediction 1a:** Given the longstanding tradition within brass instrument pedagogy of using speech syllables, and vowel tongue shapes, more specifically, as well as well-documented articulatory differences in vowel tongue shapes across languages, we predict that tongue shape during sustained note production will differ for the NZE players and Tongan players in our study. This difference will be apparent both when comparing the averages of all produced notes and when comparing groups of notes played at different intensities.
**Prediction 1b:** Because Tongan has a smaller vowel inventory than NZE, we hypothesize that Tongan vowels will have greater tongue position variability than English vowels. We predict this difference in variability will transfer to trombone performance so that Tongan players should also display higher variability in terms of the tongue positions used during trombone playing.
**Hypothesis 2:** NZE players will use a more centralized tongue position during trombone performance than Tongan players.
**Prediction 2:** Brass teachers and method books stress the necessity of keeping the vocal tract uniformly open to produce a good sound. An obvious candidate to produce such a vocal tract configuration is the neutral vowel schwa; NZE has such a vowel while Tongan does not. We hence predict that NZE players will use a more centralized tongue position during sustained note production on the trombone than Tongan players, who will assume a playing tongue position modeled on a different vowel in their native vowel system.
**Hypothesis 3:** Tongue position during trombone performance will vary with pitch.
**Prediction 3:** There is a century-old tradition within brass playing pedagogy of recommending the use of low vowels in the low register and high vowels in the high register. Based on this, we predict that the tongue positions employed during trombone performance will become increasingly closer (higher) with rising pitch.

## Materials and Methods

### Ultrasound Imaging of Speech Production and Trombone Performance

Use of ultrasound in studies with long collection times requires a method of fixing the ultrasound transducer position relative to the head; due to the lack of hard oral cavity structures in the produced images, it is otherwise impossible to directly compare images across time and/or subjects. For this study, there was the additional need to allow users to play on a trombone while having their tongue imaged with ultrasound. We used a modified version of the University of Canterbury non-metallic jaw brace (Derrick et al., 2015) that was narrow enough not to contact the trombone tubing running along the left side of player’s face. The device ties probe motion to jaw motion and thus reduces motion variance. An assessment of the motion variance of the system, evaluating tongue and head movement data collected using both ultrasound and electromagnetic articulography (Derrick et al., 2015), showed that 95 percent confidence intervals of probe motion and rotation were well within acceptable parameters described in a widely cited paper that traced head and transducer motion using an optical system (Whalen et al., 2005). We are not aware of any alternative systems available at the time of the data collection that would have been compatible with trombone performance. Similarly, electromagnetic articulography (EMA) would have been unsuitable for use in this study due to long setup times (fixing the sensors in place requires anywhere from 20 to 45 min), and the danger of sensors coming loose during a long experiment (participants were recorded for around 45 min, on average) that featured possibly more forceful tongue movements as well as higher amounts of airflow than previous speech-only experiments. Furthermore, EMA only provides data for isolated flesh points that will be inconsistently placed across individuals and it is very difficult and often impossible to position articulography sensors at the back of the tongue due to the gag reflex, meaning that we probably would have been unable to document the differences in tongue position we found at the back of the tongue using ultrasound imaging.

#### Recording Procedure

All study data were collected using a GE Healthcare Logiq e (version 11) ultrasound machine with an 8C-RS wide-band microconvex array 4.0–10.0 MHz transducer. Midsagittal videos of tongue movements were captured on either a late 2013 15′′ 2.6 GHz MacBook Pro or a late 2012 HP Elitebook 8570p laptop with a 2.8 GHz i5 processor, both running Windows 7 (64bit); the following USB inputs were encoded using the command line utility FFmpeg (FFmpeg, 2015): the video signal was transmitted using an Epiphan VGA2USB pro frame grabber, and a Sennheiser MKH 416 shotgun microphone connected to a Sound Devices LLC USBPre 2 microphone amplifier was used for the audio. The encoding formats for video were either the x264 (for video recorded on the MacBook Pro) or mjpeg codecs (for video recorded on the HP Elitebook), while audio was encoded as uncompressed 44.1 kHz mono.

Although the ultrasound machine acquired images within a 110 degree field of view at 155–181 Hz depending on scan depth (155 Hz for 10 cm, 167 Hz for 9 cm, and 181 Hz for 8 cm), the bandwidth limitations of the frame grabber meant that the frame rates recorded to the laptops reached only 58–60 Hz and were encoded in a progressive scan uyvy422 pixel format (combined YUV and alpha, 32 bits per pixel; 2:1 horizontal downsampling, no vertical downsampling) at 1024 × 768. This means that the potential temporal misalignment of image content grabbed from the top versus bottom of the ultrasound machine screen (via the frame grabber; the misalignment is called ‘tearing’) would never exceed 6.45 milliseconds.

All NZE-speaking and one Tongan participant were recorded in a small sound-attenuated room at the University of Canterbury in Christchurch, New Zealand. No equivalent room was available for the recordings of the other Tongan participants. As a result, recordings were completed in a small empty room on the campus of the Royal Tongan Police Band in Nuku’alofa, capital city of the Kingdom of Tonga.

#### Speech Elicitation

All NZE-speaking participants were asked to read a list of 803 real mono- and polysyllabic words off a computer screen, except for the first participant. Words were presented in blocks of three to five items using Microsoft PowerPoint, with the next slide appearing after a pre-specified, regular interval; the first participant read a list of words of similar length printed on paper and presented in lines of three to seven items, depending on orthographic length. Words were chosen to elicit all eleven monophthongs of NZE (see Figure 1) in stressed position plus unstressed schwa (see Heyne, 2016, pp. 252–255 for the full word list). Note that we distinguish schwa occurring in non-final and final positions in our analyses, as we were previously able to show that these sounds are acoustically and articulatory different and display phonetic variability with speech style comparable to other vowel phonemes (Heyne and Derrick, 2016a). All words were chosen to elicit all combinations with preceding coronal (/t, d, n/) and velar (/k, g/) consonants, as well as rhotics and laterals. Although it is well-known that read speech and wordlists result in somewhat unnatural speech production (Barry and Andreeva, 2001; Zimmerer, 2009; Wagner et al., 2015), this form of elicitation was chosen to ensure that the desired phoneme combinations were reliably produced, and to facilitate automatic acoustic segmentation. While the blocks usually contained words with the same stressed consonant-vowel combination, the sequence of the blocks was randomized so participants would not be able to predict the initial sound of the first word on the following slide; all NZE participants read the list in the same order. This procedure resulted in nine blocks of speech recordings lasting roughly 2 min and 20 s each, except for the first participant who was shown the next block after completing the reading of each previous block.

The same setup was used for the Tongan speakers who read through a list of 1,154 real mono- and polysyllabic words to elicit all five vowels of Tongan, both as short and long vowels, and occurring in combination with the language’s coronal and velar consonants (see Table 1; see Heyne, 2016, p. 249–251 for the full word list); all Tongan participants read the list in the same order. In Tongan, ‘stress’ is commonly realized as a pitch accent on the penultimate mora of a word (Anderson and Otsuka, 2003, 2006; Garellek and White, 2015), although there are some intricate rules for ‘stress’ shift that do not apply when lexical items are elicited via a list. We only analyzed stressed vowels with stress assigned to the penultimate mora and Tongan words are often quite short, consisting minimally of a single vowel phoneme, so it did not take as long to elicit the Tongan wordlist as the numerically shorter NZE wordlist.

Additionally, speakers from both language groups were asked to read out the syllables /tatatatata/ or /dadadadada/ at the beginning and end of each recording block to elicit coronal productions used to temporally align tongue movement with the resulting rise in the audio waveform intensity (Miller and Finch, 2011).

#### Musical Passages

The musical passages performed by all study participants were designed to elicit a large number of sustained productions of different notes within the most commonly used registers of the trombone. Notes were elicited at different intensities (*piano*, *mezzopiano*, *mezzoforte*, and *forte*; we also collected some notes produced at fortissimo intensity but removed them due to insufficient token numbers across the two language groups) and with various articulations including double-tonguing, which features a back-and-forth motion of the tongue to produce coronal and velar articulations. To control as much as possible for the intonation of the produced notes, five out of a total of seven passages did not require any slide movement and participants were asked to ‘lock’ the slide for this part of the recordings (the slide lock on a trombone prevents extension of the slide). The difficulty of the selected musical passages was quite low to ensure that even amateurs could execute them without prior practice. Participants were asked to produce the same /tatatatata/ or /dadadadada/ syllables described above at the beginning and end of each recording block in order to allow for proper audio/video alignment.

Trombone players these days can choose to perform on instruments produced by a large number of manufacturers, built of various materials and with varying physical dimensions, both of which influence the sound produced by the instrument (Pyle, 1981; Ayers et al., 1985; Carral and Campbell, 2002; Campbell et al., 2013 among others). For this reason, we asked all participants to perform on the same plastic trombone (‘pBone’ - Warwick Music, Ltd., United Kingdom) and mouthpiece (6 1/2 AL by Arnold’s and Son’s, Wiesbaden, Germany); the first English participant performed on his own ‘pBone’ using his own larger mouthpiece.

### Study Participants

Study participants were recruited through personal contacts and word-of-mouth in Christchurch and Nuku’alofa and did not receive any compensation for their participation; data collection was approved by the Human Ethics Committee at the University of Canterbury and all subjects were adults and gave written informed consent in accordance with the Declaration of Helsinki. Table 2 lists some basic demographic and other trombone-playing related information for participants in the two language groups; each group included one female player. Given the already quite restrictive criteria for inclusion in the study (playing a specific brass instrument, the trombone), we were unable to balance our sample in terms of playing proficiency; for the purpose of Table 2, playing proficiency was determined using a combination of profession (whether a player earned some (semi-professional) or most of their income (professional) by playing music) and a qualitative rating of their skill by the first author. Note that even though the Royal Tongan Police Band is a full-time professional brass band, players also serve as police officers some of the time, hence only one out of four Police Band players were rated as ‘professional.’

**TABLE 2:** Demographic data for the participants included in this study.

**Participant**	**Native**	**Age**	**Proficiency**	**Experience**
	**language**	**group**		**(years)**
S1	NZE	35–40	Professional	24
S3	NZE	65–70	Amateur	58
S5	NZE	30–35	Semi-professional	18
S12	NZE	60–65	Professional	48
S24	NZE	25–30	Intermediate	9
S25	NZE	30–35	Professional	4
S26	NZE	25–30	Professional	16
S27	NZE	30–35	Amateur	15
S29	NZE	65–70	Intermediate	58
S30	NZE	20–25	Amateur	7
	*mean:*	*40.3* ± *18*		
S4	Tongan	40–45	Amateur	28
S14	Tongan	30–35	Semi-professional	19
S15	Tongan	25–30	Semi-professional	10
S16	Tongan	30–35	Semi-professional	17
S17	Tongan	30–35	Professional	17
S18	Tongan	20–25	Amateur	7
S19	Tongan	15–20	Amateur	7
S20	Tongan	25–30	Amateur	8
S21	Tongan	15–20	Amateur	3
S22	Tongan	15–20	Amateur	7
	*mean:*	*27.2* ± *8.3*		

All NZE-speaking participants were effectively monolingual and all but two never spent significant time outside New Zealand. One participant (S30) lived in the United Kingdom for 2 years as child and spent 6 months as a High School exchange student in Germany, while one professional participant (S25) lived in the United States for 7 years and reported elementary proficiency in German and Spanish.

All except the first (S4) of the Tongan participants resided in Tonga at the time of recording and reported elementary proficiency of English acquired as part of their Tongan High School education. S4 (recorded in Christchurch) had been living in New Zealand for 20 years but spoke English with a Tongan accent and did not produce Tongan vowels that were markedly different from the other participants. Additionally, one of the players recruited in Tonga (S16) had previously spent one-and-a-half years living in Brisbane, Australia, while another (S17) reported elementary proficiency in Samoan. All remaining Tongan speakers were monolingual.

### Data Preprocessing

Audio–video misalignment resulting from recording two different USB inputs (audio and video interfaces) was resolved by aligning the tongue movement away from the alveolar region with auditory release bursts during the production of /tatatatata/ or /dadadadada/ syllables produced at the beginning and end of every recording block (see Miller and Finch, 2011).

#### Segmentation of Audio Signals

In order to automatically segment the word list recordings, we used the HTK toolkit (Young et al., 2006) as implemented in LaBB-CAT (Fromont and Hay, 2012). Phonemes matching the orthography of the input were exported from the American English version of the CELEX2 dictionary (Baayen et al., 1995) for the NZE stimuli as we were unaware of any segmentation tool available for NZE at the time. A custom dictionary was created from a Tongan dictionary (Tu’inukuafe, 1992) for all the words contained in the Tongan wordlist. All annotations were checked and corrected as necessary by the first author, with errors occurring much more frequently in the Tongan data set since the segmentation process for this data relied on an algorithm developed for speech produced in English. Three participants’ datasets recorded early on were segmented manually (two NZE and one Tongan participant).

For the musical passages, we used the Praat ‘Annotate - to TextGrid (silences)’ tool to perform a rough segmentation of the audio signal into different notes, manually corrected the boundaries, and finally applied a script to assign the appropriate label to each note from a predefined text file (Boersma and Weenink, 2014). Missed notes were eliminated, although for long sustained notes, we used a later part of the note if the participant recovered to produce a well-formed note.

#### Selection of Ultrasound Images for Articulatory Analysis

For both the NZE and Tongan data, only primarily stressed (or accented) vowels were selected for analysis; for the NZE data we used the stress markings from the New Zealand Oxford Dictionary (Kennedy and Deverson, 2005) entries, while we applied the penultimate stress/accent rule (Kuo and Vicenik, 2012) to the Tongan data. For all vowel articulations, we used the temporal midpoint to measure tongue shape, while we measured tongue shape at one third of note duration for sustained notes played on the trombone. Players of wind instruments often decrease note intensity following the beginning of notes and we wanted to make sure that we were measuring tongue shape during the steady-state of note production. For the first English participant, we manually selected a single ultrasound frame for each note as indicated by a stable tongue shape.

#### Tongue Contour Tracing and Outlier Removal

It is important to understand that ultrasound measurements are usually exported as sequences of individual images (or videos) with almost all information contained in a grainy line that represents the change of tissue density in relation to the location of an ultrasound transducer that sweeps the fan-shaped field of view in radial fashion. Although it is possible to automatically trace such images, the tools available at the time of this data collection still required a lot of manual intervention so that we decided to focus our analysis on steady-state sounds (vowels in speech and sustained notes during brass playing).

We manually traced all midsagittal tongue contours using *GetContours* (Tiede and Whalen, 2015) for MATLAB (MathWorks Inc, 2015). The tool allows the import of time stamps from Praat TextGrids and automatically interpolates a minimum of three anchors placed manually to a cubic spline of 100 points length outlining the tongue shape produced in each individual ultrasound frame. Once all vowel or note tokens were traced for a certain participant, we employed various search terms to assemble all tokens for a specific stressed/accented vowel or note into a separate data set based on the information contained in the TextGrid imported from Praat; a small number of visual outliers (around 1% of tokens for speech and 1.4% for notes) were subsequently removed by plotting tokens of the same vowel or note together. Note that although Tongan distinguishes short and long vowels (often analyzed as one or two morae, respectively, see Feldman, 1978; Kuo and Vicenik, 2012), the articulatory differences between these phonemes are very small, and we thus decided to treat these tokens as a single underlying motor target. Overall, the models reported below were estimated based on 12,256 individual tongue contours of vowel tokens (7,834 for NZE, 4,422 for Tongan) and 7,428 tongue contours of sustained note production (3,715 for NZE, 3,713 for Tongan). Full token numbers are included in the R notebooks available on GitHub^1^.

Due to variable image quality and the unconstrained placement of GetContours anchors on each ultrasound frame, individual tokens differed greatly in length. Generalized additive mixed models (GAMMs) provide appropriate confidence intervals for noisy data, eliminating the necessity of cropping data, and are able to handle input data of different lengths by modeling individual differences similar to (linear) mixed effects models. Missing data points are replaced with an average value of existing data in the same position, taking individual variability, as well as variability inherent to the currently observed condition, into account. Nonetheless, we did remove a few tokens occurring in specific contexts (e.g., a certain note produced at fortissimo intensity, as mentioned above) where we did not have a sufficient number of tokens for each language group to estimate reliable average tongue shapes.

#### Rotating and Scaling Ultrasound Traces Across Individuals

Our research question necessitated the direct comparison of articulatory data across different vocal tract activities and individuals. Ultrasound data are particularly difficult in this regard since no anatomical landmarks are visible in the recorded images, and tongue shape during speech production can furthermore vary with individual differences in vocal tract shape and biomechanics (Simpson, 2001, 2002; Fuchs et al., 2008; Brunner et al., 2009; Rudy and Yunusova, 2013; Lammert et al., 2013a, b; Perrier and Winkler, 2015). Various methods have been developed for determining and comparing, e.g., the curvature of selected tongue shapes (Ménard et al., 2011; Stolar and Gick, 2013; Zharkova, 2013a, b; Dawson et al., 2016) or the relative articulatory height and fronting of a certain vowel tongue shape (Lawson and Mills, 2014; Noiray et al., 2014; Lawson et al., 2015), independent of anatomical landmarks. For the purposes of this study, however, we needed to compare information regarding both tongue shape and relative position, so we decided to transform all data into a common space prior to our statistical analysis. Across both language groups and the different vocal tract behaviors, the high front vowel /i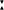/ appeared to be most constrained by individual vocal tract morphology – and previous research has shown /i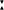/ to have a relatively stable production pattern across languages (Chung et al., 2012). According to Chung et al. (2012) in terms of rotational differences, cross-linguistic differences were mostly due to a more back location of /a/ and a more fronted location of /u/ produced by English and Japanese speakers relative to those of the three other languages.

**FIGURE 2:**
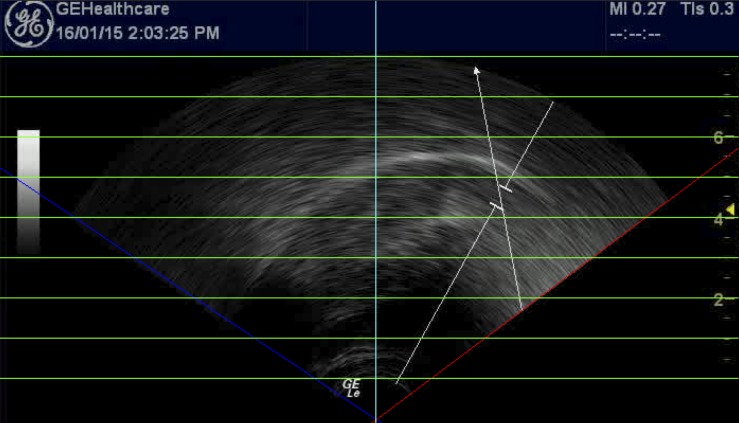
Estimation of the ‘virtual origin’ and pixel scale from a randomly selected ultrasound image by overlaying various lines (slanted blue and red lines delineate the fan-shaped scan area, green horizontal lines replicate the scan depth indicators at right-hand side of the image, and the turquoise vertical line serves as perpendicular reference).

**FIGURE 3:**
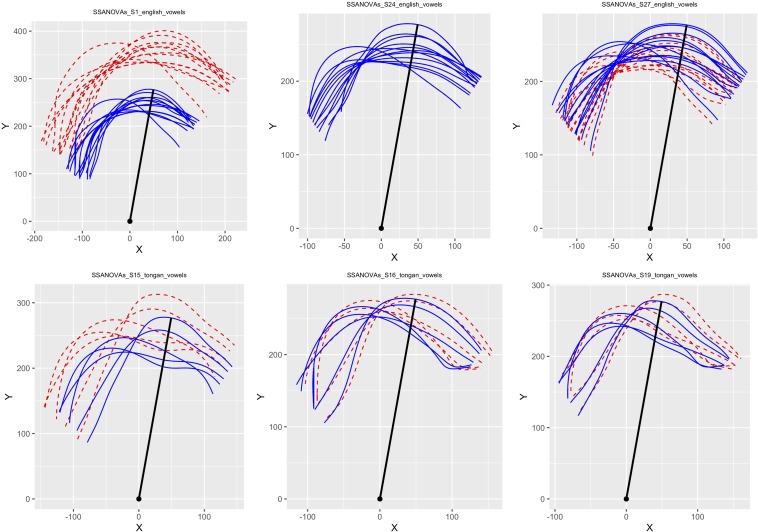
Plots illustrating the effects of the rotation and scaling procedures on the SSANOVA average curves for monophthong productions by three NZE **(top row)** and three Tongan participants **(bottom row)**. The units on the *x*- and *y*-axes represent pixel distances relative to the estimated virtual origin and differed across subjects due to vocal tract size and ultrasound machine depth setting. S24 **(middle of top row)** featured the smallest scan depth setting and was thus chosen to provide the target vector for the rotation and scaling procedures, shown as a black radial line extending from the virtual origin in each plot. Adapted from Heyne (2016, p. 154).

For both NZE and Tongan, each subject’s ultrasound contours were rotated to align the position of the (mean) average contour’s highest points during the production of the high front vowel /i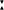/ (FLEECE in NZE). Note that for NZE participant S1, the highest point actually occurred during the averaged productions of /e/ (the NZE DRESS vowel) and we used this location instead; this articulatory reversal may either have been due to the extremely close articulation of the DRESS vowel (/e/) in modern NZE (cf. Introduction), possibly interacting with the increasing diphthongization of the FLEECE vowel (/i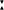/; cf. Maclagan and Hay, 2007), or the speaker could be a ‘flipper’ as suggested by Noiray et al. (2014; see also Ladefoged et al., 1972). Contour height was measured by calculating the distance of each point in relation to the virtual origin of the ultrasound signal. Figure 2 illustrates the procedure used to identify the virtual origin on a sample ultrasound image. The Figure also shows green lines that can be used to convert image pixel values to real-life distances (cf. Heyne and Derrick, 2015c). (Note that all our articulatory images have the tongue tip at the right.) Identifying these two locations allowed us to calculate a two-dimensional vector connecting the two locations, which in turn was used to rotate tongue traces in polar space without affecting the underlying variability. Each set of contours was also scaled so that the furthest point of the high front vowels lined up to that of S24 NZE, who had the overall smallest vocal tract and hence served as the target space for all other data (vowel and playing contours across both language groups). Figure 3 shows the scaling applied to six participant data sets in our study.

We also used the ‘virtual origin’ to correct one participant’s data (S12 NZE) for whom the ultrasound transducer seemed to have moved partway through the recording session. Although the overall quality of palate shapes collected at regular intervals during the experiment by tracing tongue movement during water swallows (cf. Epstein and Stone, 2005) was insufficient for inter-subject alignment, the availability of such traces for the particular participant greatly helped in determining the required amount of rotation and translation to correct for the transducer movement; we were also able to confirm the temporal location of transducer movement by examining video of the participant’s face collected throughout the recording session (cf. Heyne, 2016, pp. 144–145).

### Statistical Analyses

The x- and y-coordinates of all tongue traces along with vowel identity and phonetic context for the proceeding and following speech tokens, and note identity (pitch) as well as intensity (loudness) for the five different trombone notes, were transferred to R (version 3.5.1, R Core Team, 2018) and transposed into polar coordinates using the virtual origin coordinates for the participant with the smallest vocal tract. To test each prediction, we generated Auto-Regressive Generalized Additive Mixed Models (GAMMs) using the bam function from the package mgcv (Wood, 2011, 2015).

For model back-fitting, we started by visually evaluating the patterns in the data and ran various models (e.g., no random effects, random effects, multiple predictors including sex and playing proficiency, etc.). However, when exploring the data visually, it was apparent that the differences between speakers, and how they produced notes at varying intensities are captured by the optimal model. The *R*^2^ value of the optimal model is more than double that of the model without random effects. Using visual inspection, the *R*^2^ values allowed us to select the optimal model.^2^ Once the optimal model was obtained, we estimated the correlation level in the residuals and generated a new model that took the autocorrelation in the residuals into account. We also performed a well-formedness test using the gam.check function from the mgcv package to inspect the residuals themselves and determine whether the value *k* = *10*, referring to the number of knots defining the smoothing spline basis function was sufficient.

The resulting model for Hypothesis 1 is shown in formula 1 below. This and all subsequent model formulae employ standard mgcv syntax defined as follows: s = smooth term used to estimate the curvature of tongue contours; bs = basis function of the smooth term; cr = cubic regression spline; fs = factor smooth that allows the estimation of interaction smooths for random effects; k = number of knots to control for the degree of non-linearity in the smooth; by = used to model non-linear interactions between a factor and the predictor; m = *n*, e.g., 1, parameter specifying how the smoothing penalty is to be applied, allowing the shrinkage toward the mean for the random effects; more details can be found in Wood (2011, 2017) and Sóskuthy (2017).

1: rho ∼ LanguageNoteIntensity + s(theta, bs = “cr”, k = 10) + s(theta, k = 10, bs = “cr”, by = LanguageNoteIntensity) + s(theta, subject, bs = “fs”, k = 10, m = 1, by = NoteIntensity)

Where *rho* is the distance of the fitted tongue contour point from the virtual origin, and *theta* is the angle in relation to the virtual origin. The variable *LanguageNoteIntensity* encodes the interaction between language (Tongan, NZE), note identity (Bb2, F3, Bb3, D4, F4), and note intensity (*piano*, *mezzopiano*, *mezzoforte*, *forte*). It is used as a fixed effect and as a contour adjustment. The variable *NoteIntensity* encodes the interaction of note identity and note intensity. It is used as a contour adjustment for the random effect that uses *subject* ID, used to model the within-speaker variations with respect to note productions. All variables were ordered to allow for a meaningful interpretation of the smooths.

For Hypothesis 1, prediction 1b required the variance to be analyzed rather than position itself. For this model, the source data was summarized by grouping ultrasound theta angles into 100 bins, and computing variance of tongue position for each bin by speaker and token (both musical notes and vowels). In all other ways, the formula was derived as for predictions 1a, 2, and 3. The resulting model is shown in formula 2:

2: var(rho) ∼ LanguageNote + s(theta, bs = “cr”, k = 10) + s(theta, bs = “cr”, k = 10, by = LanguageNote) + s(theta, subject, bs = “fs”, k = 10, m = 1, by = LanguageNote)

This formula uses *var(rho)* for the variation in the distance of the fitted tongue contour point from the virtual origin, and *LanguageNote* is the ordered interaction of language (Tongan, NZE), and notes/vowels. As with formula 1, all variables were ordered to allow for a meaningful interpretation of the smooths.

For Hypothesis 2 (separate models run for NZE & Tongan), the resulting model is shown in formula 3:

3: rho ∼ Token + s(theta, bs = “cr”, k = 10) + s(theta, k = 10, bs = “cr”, by = Token) + s(theta, subjectToken, bs = “fs”, k = 10, m = 1) + s(theta, precedingSoundToken, bs = “fs”, k = 10, m = 1) + s(theta, followingSoundToken, bs = “fs”, k = 10, m = 1)

Similar methods were used for Hypothesis 2 and the related predictions (i.e., note-by-vowel and note-by-note differences). We used the relevant notes and vowels as fixed effects (Token in formula 2) and as tongue contour adjustment (using a ‘by’ specification). In addition, we specified three random effects. We created a new variable forming an interaction between the subject producing each given note or vowel quality (*subjectToken*); this variable was used as our first random effect. The two additional random effects were the interaction between the preceding sound, following sound, and vowel identity (and note intensity by note identity for notes, i.e., *precedingSoundToken* and *followingSoundToken*). These random effects allowed us to fine-tune the analysis to account for subject and contextual differences. All variables were ordered again to allow for a meaningful interpretation of the smooths. We performed the same back-fit and well-formedness analyses as for hypothesis 1.

For all three models, we used custom functions (Heyne, 2019) to visualize the predictions from our models in polar coordinates, using the package plotly (Sievert et al., 2017) to plot the transformed outputs of the plot_smooth function from the package itsadug (Van Rij et al., 2015). Additionally, we used the function plot_diff from the latter package to determine the intervals of significant differences for the whole range of given data points (in our case, the whole midsagittal tongue contour from the front to the back of the tongue) and added these as shaded intervals to our polar plots. All our analyses in the form of R notebooks are available on GitHub^3^.

## Results

### Prediction 1a: Tongue Position During Sustained Note Production Will Differ for NZE Players and Tongan Players

Our final model investigating overall language differences during trombone performance found a robust interval of significant difference at the back of tongue with NZE players utilizing a more retracted tongue position. The model also showed a difference at the front of tongue where the Tongan players use a more elevated position, however, this difference was not very reliable across comparisons involving different notes produced at different intensities.

The full details (including a model summary) for the final GAMM investigating whether tongue shape during trombone playing differs across the two language groups included in this study are available as part of our supplementary notebooks on GitHub^4^. The final model includes an autocorrelation model to account for massive amounts of autocorrelation observed in the residuals of the same model that did not account for autocorrelation (see Sóskuthy, 2017, section 2.3; Wieling, 2018, section 4.8 for discussion). All plots included in this paper are based on models estimated using fast model estimation via fREML (fast restricted estimate of maximum likelihood) in combination with the discrete = TRUE flag. Our final model had an *R*^2^ value of 0.855 and used 742,800 data points (7,428 individual tongue contours).

**FIGURE 4:**
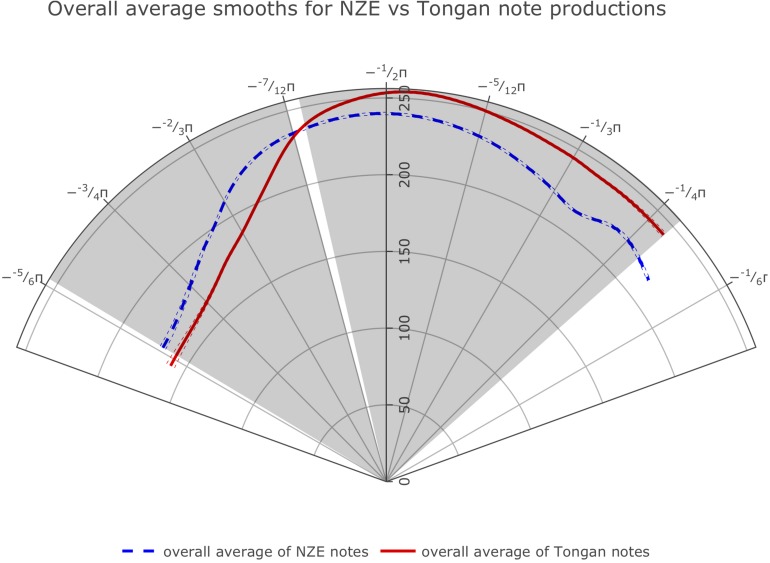
Average smoothing splines for all notes produced by the players from each language group fit on predicted values from our GAMM model investigating whether tongue shape during trombone playing differs across the two language groups. Angular values (Theta) are specified in radians as fractions of Pi, while radial values (Rho) are expressed as pixel values in reference to the participant with the smallest vocal tract. We show the front of the tongue at the right and the back of the tongue at the left throughout this paper.

The optimal model design prevented us from directly comparing the average smooths for all notes produced by the players from each language group. However, we were able to fit smoothing splines of the average tongue shapes used across all notes and intensities across the two language groups by getting predicted values for all ingoing data points from our GAMM model using the predict.bam function from the mgcv package and fitting smoothing splines on these data split up by native language using R’s generic predict.smooth.spline function. The overall average splines (Figure 4) show clear differences at the back and at the front.

**FIGURE 5:**
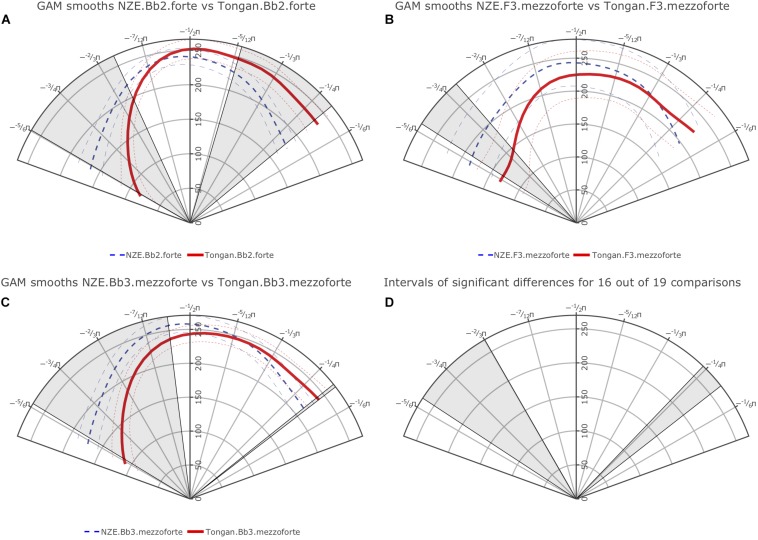
**(A)** top left: Plot of smooths estimated by our first GAMM for the note Bb2 played at *forte* intensity for the NZE (blue, dashed line) vs. Tongan participants (red, solid line); **(B)** top right: Smooths for the note F3 produced at *mezzoforte* intensity; **(C)** bottom left: Smooths for the note Bb3 produced at *mezzoforte* intensity; **(D)** bottom right: Overlap of significant differences among 16 out of 19 note comparisons (roughly 84.2% agreement, compare Table 3).

Additionally, we carried out pairwise comparisons for each note at the four different intensities (*piano*, *mezzopiano*, *mezzoforte*, *forte*) across the language groups and all individual comparisons show at least one interval of significant difference (either at the back or the front of the tongue). Figure 5 provides plots of the smooths estimated for each language group for the notes Bb2 at *forte* intensity, F3 at *mezzoforte* intensity, and Bb3 at *mezzoforte* intensity with areas of significant difference indicated by shading; the comparisons for F3 and Bb3 at *mezzoforte* intensity feature the largest token numbers in our data set (1,089/1,169 tokens for F3 and 986/1,042 tokens for Bb3 for NZE and Tongan, respectively). Note that overlap of the 95% confidence intervals is an imprecise diagnostic of significance differences between portions of two smooths. Instead, the shaded intervals, indicating regions of significant difference, have been determined using the precise and accurate statistical procedure implemented via the plot_diff function from the itsadug package.

Overall, we find robust differences at the back of tongue (area from roughly -3/4π to -2/3π as shown in the plots) for all individual note comparisons except for the notes Bb2 produced at *piano* intensity, and D4 at *mezzopiano* intensity (the interval of significant differences for F3 at *mezzoforte* intensity barely extends past -3/4π but nonetheless seems substantial). Toward the front of the tongue, our plots also show significant differences for most comparisons, indicating a more elevated position used by the Tongan players; differences at the front of the tongue consistently occur at *forte* intensity but are notably absent for 2 out of 5 comparisons (notes F3 and D4) at *mezzoforte* intensity where we have substantial token numbers (1,089/1,169 tokens for F3 and 368/385 tokens for D4 for NZE and Tongan, respectively). However, we should not assign too much weight to any differences occurring past −1/3π at the front of the tongue and −3/4π at the back of the tongue due to the fact that we are averaging across subject data with different trace lengths that were normalized by rotation and scaling (see section “Rotating and Scaling Ultrasound Traces Across Individuals” above). Additionally, when overlaying the areas of significant differences for all individual comparisons the agreement becomes very small at the front of the tongue while 16 out of 19 comparisons (84.2%) show the substantial difference noted for the back of the tongue (see Figure 5D). Table 3 provides a list of the intervals of significant differences for all individual note comparisons.

**TABLE 3:** Intervals of significant difference for all note comparisons.

	**Interval at front**		**Interval at back**	
	**of the tongue**		**of the tongue**	
Bb2, forte	−2.65	−1.96	−1.32	−0.65
Bb2, mezzoforte	−2.65	−2.06	−1.30	−0.65
Bb2, mezzopiano	−2.39	−1.98	−1.20	−0.82
Bb2, piano	NA	NA	−1.34	−0.65
Bb3, forte	−2.65	−1.92	−1.26	−0.65
Bb3, mezzoforte	−2.65	−1.64	−0.67	−0.65
Bb3, mezzopiano	−2.65	−1.98	−1.02	−0.65
Bb3, piano	−2.65	−1.94	−0.82	−0.65
D4, forte	−2.65	−1.92	−1.30	−0.65
D4, mezzoforte	−2.65	−1.74	NA	NA
D4, mezzopiano	NA	NA	−1.48	−0.65
D4, piano	−2.65	−1.92	−1.32	−0.65
F3, forte	−2.65	−1.94	−1.20	−0.65
F3, mezzoforte	−2.59	−2.27	NA	NA
F3, mezzopiano	−2.65	−1.92	−1.26	−0.65
F3, piano	−2.65	−2.06	−1.56	−0.65
F4, forte	−2.65	−1.80	−1.16	−0.65
F4, mezzoforte	−2.65	−1.76	−1.16	−0.65
F4, piano	−2.61	−1.96	−1.20	−0.65
Agreement 16/19 comparisons	−2.59	−2.06	−0.82	−0.65

*Comparisons are based on variable token numbers and there were no tokens of F4 produced at *mezzopiano* intensity by the NZE players, hence we were unable to carry out a comparison for that note. Note also that regrettably the ‘scatterpolar’ plotting modality from the plotly R package requires input values scaled in degrees. We thus had to apply a transformation to get our values to show up correctly but were able to overlay a scale in radians using fractions of π; these are equivalent to the following numbers: −5/6π = −2.62; −3/4π = −2.36; −2/3π = −2.09; −1/2π = −1.57; −5/12π = −1.31; −1/3π = −1.05; −1/4π = −0.79; −1/6π = −0.52.*

### Prediction 1b: Tongan Vowels Will Have Greater Production Variability Than English Vowels

The full details (including a model summary) for the final GAMM describing the difference in variance for tongue position distance from the virtual origin along the tongue curvature between NZE and Tongan are again available as part of our supplementary notebooks on GitHub^4^. Our final model had an *R*^2^ value of 0.863 from 12,704 data points from 180 variance curves.

**FIGURE 6:**
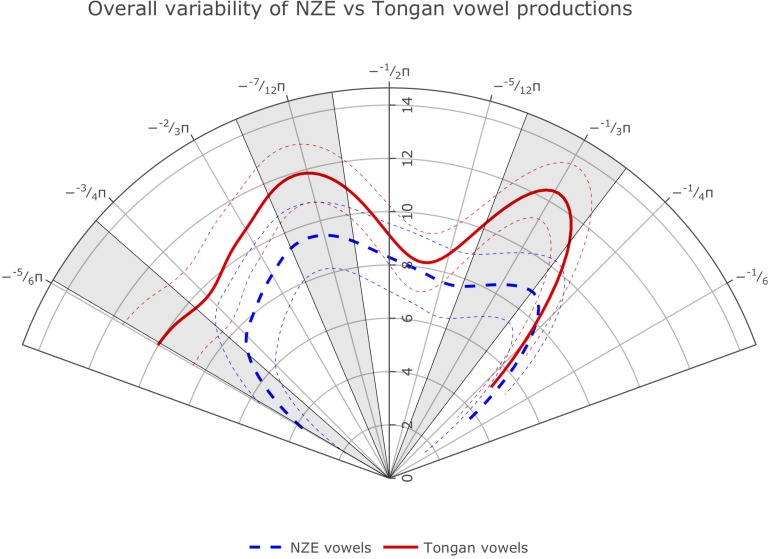
Average smoothing splines for variance in tongue surface distance from the ultrasound virtual origin for NZE and Tongan vowel productions.

The optimal model design prevented us from directly comparing the average smooths for the variance of each language group. However, we were able to fit smoothing splines of the average tongue position variance used across vowels by participants from both language groups by getting predicted values for all ingoing data points from our GAMM model, similar to the method used when addressing Prediction 1a. The overall average splines (Figure 6) show clear variance differences at portions of the front, middle, and back of the tongue, indicating that the Tongan participants’ vowel productions were more variable than those produced by the NZE speakers.

We also carried out the same comparison for NZE and Tongan note productions and found that Tongan trombone notes show more variability than English trombone notes for a small portion of the tongue surface between −2/3 and −7/12 π radians. These results had an *R*^2^ value of 0.817 from 6,446 data points from 100 variance curves. Supplementary Figure S1 can be found in the Supplementary Material.

### Prediction 2: NZE Players Will Use a More Centralized Tongue Position During Trombone Performance Than Tongan Players

The full details for the two final GAMMs describing the relationship of note tongue contours to vowel tongue positions in the two languages are also available on GitHub^4^. The final model for NZE had an *R*^2^ value of 0.852 and used 1,154,900 data points (11,549 individual tongue contours), while the final model for Tongan had an *R*^2^ value of 0.898 and used 813,500 data points (8,135 individual tongue contours).

**FIGURE 7:**
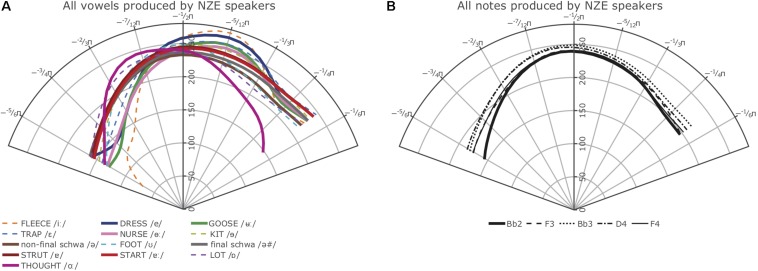
**(A)** Left: Average smooths for the NZE monophthongs produced by all NZE speakers included in this study. **(B)** Right: Average smooths for the five different notes produced by the NZE-speaking trombonists.

**FIGURE 8:**
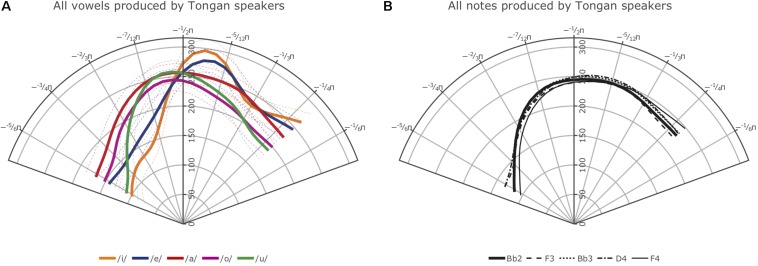
**(A)** Left: Average smooths for the Tongan vowels produced by all Tongan speakers included in this study; thin dashed lines represent 95% confidence intervals. **(B)** Right: Average smooths for the five different notes produced by the Tongan-speaking trombonists.

Figures 7, 8 show the smooths for all vowels (A) and notes (B) produced by the participants in the two language groups. The confidence intervals plotted with the Tongan vowels in Figure 8A, although not as appropriate as the intervals estimated using the plot_diff function shown in our plots addressing Hypothesis 1, indicate that even in a language with a small vowel system, the average vowel tongue shapes overlap considerably so they are not statistically different in terms of their articulation when properly accounting for variance such as subject-specific productions and preceding and following phonemes. Note that we decided not to include the 95% confidence intervals for the NZE vowels in Figure 7A as the crowded NZE vowel spacealready makes the left panel of the Figure very hard to read; for the same reasons, no confidence intervals are shown with the note smooths (Figures 7B, 8B).

**FIGURE 9:**
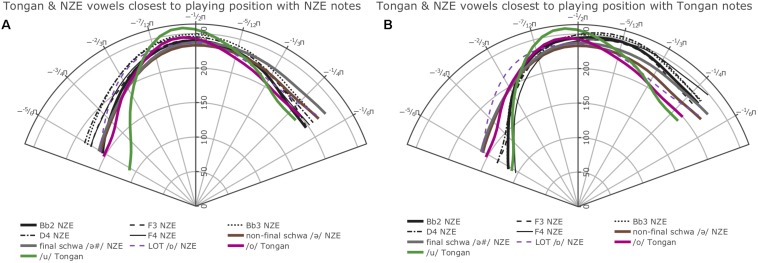
Plots showing the GAMM smooths for the vowels most closely approximated by note contours produced **(A)** by the NZE participants, **(B)** by the Tongan participants.

While inspection of all individual smooths comparisons from our models (see R notebooks on GitHub^5^) indicated that the tongue shapes employed by the NZE players pattern somewhat closely with up to seven different monophthongs in NZE (KIT /ɘ/, non-final-schwa /Ə /, FOOT /Ω/, final schwa /Ə #/, STRUT /ɐ /, START /ɐ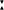/, and LOT /ɒ/), the closest match seems to be with both the vowels occurring in the word ‘lot’ (LOT /ɒ/) and the neutral vowel schwa when it occurs in final position (/Ə #/); note that NZE being non-rhotic, the latter group also includes words ending in -er such as ‘father.’ In Tongan, in contrast, we do not find such a close match and the vowel tongue shape most closely approximated during trombone playing seems to be that for the vowel /o/; however, this is only the case visually – the vowel /u/ actually features less intervals of significant differences to the tongue shapes assumed during sustained notes produced by the Tongan players. We also see some consistent patterning with the vowel /a/ at the front of tongue. Nonetheless, all individual comparisons between these three vowels and the average note productions feature at least one interval of significant difference, indicating that the match between vowel and note tongue shapes is much closer in Tongan than in NZE. All of the closest-matching vowels identified for Tongan differ from NZE LOT and schwa (produced in both non-final and final environments) mostly in terms of tongue retraction. Figure 9 shows plots of the vowels in both languages most closely approximated by the respective players’ note productions. The left panel overlays the NZE players’ note tongue contours, while the right panel does the same with the Tongan players’ note contours.

**FIGURE 10:**
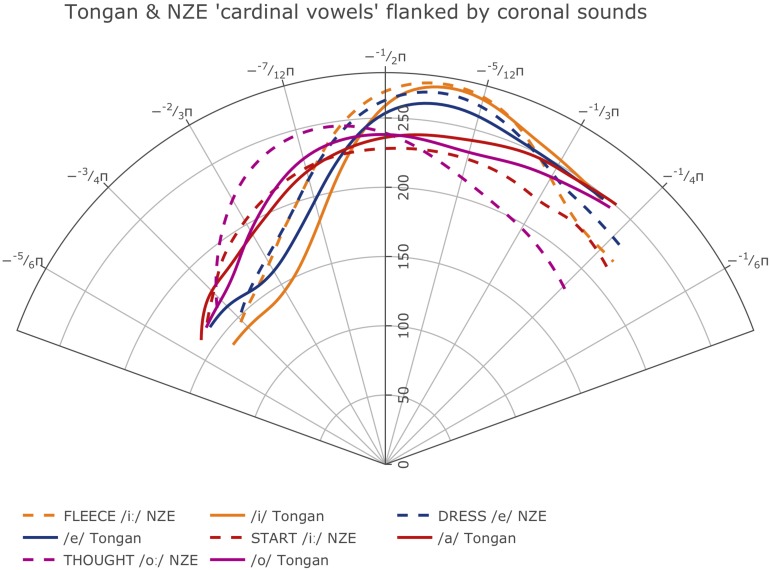
Average tongue contours for selected monophthongs produced by participants from the two different language groups when flanked by coronal consonants (Tongan = solid lines, NZE = dashed lines); the Figure includes only vowels we would expect to be roughly similar across the two languages in terms of their acoustics.

While the average tongue shapes during sustained trombone note production are clearly different for the two language groups as shown in Figure 4 (cf. also comparisons of selected notes and intensities in Figure 5), average tongue contours for a subset of monophthongs of both languages that can be expected to feature relatively similar articulations across the two languages (based on their acoustic descriptions), map up fairly well when regarded in a controlled phonetic environment, as shown in Figure 10. Note that in each case, the NZE vowel articulations feature a more retracted tongue shape than the one used by the Tongan participants, in agreement with the overall differences observed at the back of the tongue during note productions. Acoustic descriptions of NZE (Gordon et al., 2004; Maclagan and Hay, 2004; Bauer et al., 2007; Bauer and Warren, 2008) indicate that NZE DRESS (/e/) is ‘close’ compared to a more ‘cardinal’ pronunciation of the /e/ vowel in Tongan; similarly, the NZE THOUGHT vowel (/o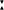/) is comparatively raised, possibly due to a chain shift documented for other varieties of English that motivates it to move into the space vacated by the fronted GOOSE vowel (/ʉ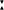/) (Ferragne and Pellegrino, 2010, p. 30; cf. Scobbie et al., 2012; Stuart-Smith et al., 2015).

### Prediction 3: The Tongue Positions Employed During Trombone Performance Will Become Increasingly Closer (Higher) With Rising Pitch

The right-hand panels (B) of Figures 7, 8 show the smooths for the different notes produced by the players from the two language groups. While the NZE players as a group display a more-or-less consistent pattern of using a higher tongue position for higher pitch notes (except for the note F4), this pattern does not apply to the Tongan group. Instead, we find that in the area where we might expect the narrowest vocal tract constriction, the highest tongue contour is that of the lowest included note, Bb2, while D4 represents the highest tongue contour anterior of this location. Overall, upon visual inspection of the smooths and tabulation of intervals of significant differences for all notes at different intensities (produced in the same manner as Table 3 above), we observe the biggest differences between notes produced at *mezzoforte* which may be specific to this intensity level but could also be an artifact of having larger token numbers at *mezzoforte*. The reader is also encouraged to view the parametric plots on GitHub^6^; these plots show a clear difference with respect to the overall differences in the parametric terms (fixed effects) and how variable they are in both NZE and Tongan on the one hand, and in the position of the notes on the other. Higher notes (produced at louder intensities) seem to show a higher tongue position compared to lower notes; NZE shows an overall lower tongue position compared to Tongan in lower notes and a comparable position in the higher notes.

Out of total 76 note comparisons (40 for Tongan, 36 for NZE due to missing tokens for the note F4 produced at *mezzopiano* intensity), only 11 featured significant differences at either the back or front of the tongue (none had both). For Tongan these were: Bb2 vs. F4, D4 vs. F4, and Bb3 vs. F4 at *mezzopiano* intensity, and Bb3 vs. F4 and F3 vs. F4 at *piano* intensity; note that each comparison involved the note F4 for which we have the smallest token numbers. For NZE these were: Bb2 vs. Bb3, Bb2 vs. D4, and Bb3 vs. D4 at *forte* intensity, Bb2 vs. Bb3 and Bb2 vs. D4 at *mezzoforte* intensity, and Bb2 vs. F4 at *piano* intensity.

## Discussion

In this paper, we have presented a comprehensive analysis of midsagittal ultrasound data that has allowed us to investigate a number of questions regarding the relationship between speech production and brass instrument performance, and some longstanding assumptions propagated by teachers of brass instruments whereby the tongue shapes assumed during performance resemble those employed during speech production, especially when producing vowels. We compared average tongue shapes of vowel articulation and tongue positioning during trombone performance estimated based on large token numbers using generalized additive models, a statistical technique that properly accounts for contextual factors and unknown variability such as speaker/performer idiosyncrasies. As far as we know, this article also presents the first comprehensive articulatory descriptions of both the New Zealand English and Tongan vowel systems. In the following, we evaluate our hypotheses and specific predictions based on the results presented in the previous sections and discuss some other constraints affecting tongue shape during brass instrument performance.

### Hypothesis 1, Prediction 1a: Language Influence on Trombone Performance

Our data provide clear support for our first hypothesis, prediction 1a, whereby a brass player’s language will influence the vocal tract states they assume during performance on their instrument. We observed significant differences at the back of the tongue across our two language groups made up of NZE and Tongan speakers both overall as well as for 16 out of 19 individual note comparisons. These comparisons encompassed five different pitches performed within the standard playing range of the trombone at soft (*piano*) to loud (*forte*) intensities. All comparisons featured at least one interval of significant differences (either at the back or front of tongue), providing strong support for our Prediction 1a which stated: Tongue position during sustained note production will differ for NZE players and Tongan players, both overall and when comparing individual notes played at different intensities. However, there also seem to exist a lot of other factors influencing midsagittal tongue shape during trombone performance (e.g., airflow requirements and the potential of vocal tract resonances influencing the produced sound) – we will return to those later on in the discussion.

### Hypothesis 1, Prediction 1b: Language and Token Position Variability

Our data provides support for our first hypothesis, prediction 1b, whereby tongue position variability in vowel production will be related to the segmental inventory size of the language. Tongan has fewer vowels than NZE, and so it was predicted to have higher token variability. The results show higher average Tongan vowel production variability in the contour comparison for portions of the tongue front, middle, and back (Figure 6). Tongue position variability differences between Tongan and NZE extend along the entire surface of the tongue. Therefore, the results provide direct support for dispersion theory (Liljencrants and Lindblom, 1972; Lindblom, 1986; Al-Tamimi and Ferragne, 2005).

The results also suggest that this dispersion might extend to note productions on the trombone, as these were also more variable for the Tongan participants; however, this was true only for a small portion at the back of the tongue and as such, is not a strong effect (see Supplementary Figure S1). Moreover, significant differences are not visible in any comparison of specific notes or vowels, probably because these measures are based on single variance averages by participant, which is a very low number for GAMMs.

The note results in the Supplementary Figure S1 also show that note variability is similar to vowel variability along the full length of the imaged tongue contours; however, the Tongan contours for notes do not extend quite as far back as the data for the NZE participants, which, however, has no meaningful impact on the interpretation of Figure 4.

### Hypothesis 2: Use of a Schwa-Like Vowel Shape by the NZE Players

With Hypothesis 2, we were trying to determine whether an articulatorily informed interpretation of a popular recommendation among brass players, namely, to keep the vocal tract ‘open’ to produce a good sound, would be supported by empirical data. Various studies (see Heyne and Derrick, 2016b) have provided ambiguous evidence regarding the openness of the vocal tract, mostly presenting data for the oral cavity (but see section “Other Constraints on Tongue Shape During Brass Instrument Performance” below for some findings regarding glottis opening during brass instrument performance) and often interpreting their results in comparison to vowel tongue shapes which we will address in more detail below. We specifically predicted that the average tongue shape assumed during trombone playing by the NZE-speaking participants in our study would approximate the vowel tongue shape for the neutral vowel schwa while the Tongan players would assume a different shape as their native language does not contain a neutral vowel such as schwa. Indeed, we found that for the NZE players, two out of the three vowel tongue shapes most closely approximated by their playing tongue shapes were schwa when produced in non-final and final environments. However, the only NZE vowel that showed no significant intervals of difference to the NZE note tongue shapes for any comparisons was LOT (/ɒ/), hence our Prediction is not fully supported. In terms of the note tongue shape assumed by the Tongan players, the data support our prediction in that they clearly use a more ‘centralized’ tongue shape during playing; the most salient difference, however, seems to occur at the back of the tongue and we will return to this point later on.

### Hypothesis 3: Tongue Position During Note Production and Its Relation to Pitch

Our models fit on the full data set also allowed us to probe a longstanding assumption within brass pedagogy, namely that players should raise their tongue when ascending throughout a brass instrument’s register. More precisely, many brass method books published from the 19th century onward recommend the use of low vowels in the low register with a gradual change toward high vowels to be employed when playing in the high register. Our prediction 3 represents a less strong version of such claims whereby we simply predicted that the tongue shapes assumed during sustained note production would become increasingly closer with rising pitch. The results presented in sections “Prediction 2: NZE Players Will Use a More Centralized Tongue Position During Trombone Performance Than Tongan Players” and “Prediction 3: The Tongue Positions Employed During Trombone Performance Will Become Increasingly Closer (Higher) With Rising Pitch” above do not provide much support for this prediction: while there is some indication of NZE players using a higher tongue position for higher notes, this pattern is much less clear for the Tongan participants. Additionally, none of the vowels typically mentioned in brass method books (e.g., /o/ to /i/) seem to map up particularly well with note tongue shapes used by the NZE players in our study, although the vowel tongue shape might be approximated by players who speak native languages that do not have a neutral/central vowel such as schwa. In addition to Tongan investigated in this study, similar considerations apply to languages like Spanish and Japanese.

Note however, that in the first author’s more recent work using real-time MRI of the vocal tract to record tongue movements during trombone performance (Iltis et al., 2019) there is clear evidence for tongue raising in the midsagittal (and coronal) planes with ascending pitch. Hence we might speculate that the lack of pronounced differences in the ultrasound data presented here may be related to the use of a jaw brace for ultrasound transducer stabilization that ties tongue motion to jaw position.

### What Is a Possible Mechanism for Language Influence on Brass Instrument Performance?

Having established that there are significant differences regarding the midsagittal tongue shape used by players from the two different language groups investigated in this study, we may now move on to speculate what a possible mechanism for such a relationship might look like. Articulatory Phonology (Browman and Goldstein, 1986, 1992; Goldstein and Fowler, 2003) posits that phonological units of speech can be analyzed as constrictions occurring at various locations along the vocal tract, and we suggested in the introduction that these gestures might take the form of motor memory when being transferred across different vocal tract activities. Since we observed a far from complete overlap of midsagittal tongue shapes during speech and trombone performance (even for the NZE players), and there may as well be other differences that we cannot measure with midsagittal ultrasound images (jaw opening, coronal tongue shape), we need to explore in more detail how vowel gestures from speech production might transfer to brass playing.

It has previously been shown that the tongue can be divided into at least four independent sections (along the sagittal plane) within the oral cavity (Stone et al., 2004) and it is possible that, for example, during brass playing, tongue root retraction forms an important vocal tract constriction that affects airflow and tone color (more below). In this vein, we might think of learning to play a brass instrument as a process whereby multiple vocal tract gestures relevant to this activity have to be fine-tuned in order to achieve a good sound, as well as flexibility in being able to change, and articulate, various notes. Tongue shape during brass playing might be determined by local optimization processes applying to various parameters including vocal tract constrictions based on gestures already encoded in the system as motor memory. The latter case, of course, is where we suggest vowel tongue shapes would come in. Note that we regard this process as local, rather than global, optimization in agreement with Loeb (2012) who argues that “good-enough strategies” such as trial-and-error learning will lead to “a diversity of solutions that offers robustness for the individual organism and its evolution” (p. 757; see Ganesh et al., 2010, for empirical evidence of local optimization during motor learning).

In contrast to a theory of optimal control, a theory of local optimization is in agreement with the astonishing amount of individual variability observed in this and earlier empirical studies on brass playing (and speech production, for that matter) and offers a plausible account of how the language differences we observed may arise. Imagine that a beginning player might initially explore different local optima (different vowel tongue shapes but possibly also language-unrelated gestures such as the tongue configuration used during whistling) before settling on a more stable default tongue shape that would be locally optimized using acoustic information and effort minimization. Using a vowel tongue shape as starting point would seem to reduce both error and the required effort, at least until the player develops sufficient motor memory for the new motor action. In turn, it should also be possible to gradually ‘unlearn’ (cf. Heyne and Derrick, 2015b, p. 7) language-related tongue shapes by developing brass playing-specific motor memory, reducing language influence on brass playing among highly skilled performers.

### Articulatory Setting Theory

Another possible mechanism for language influence on brass instrument performance is provided by the concept of language-specific articulatory settings (cf. Wallis, 1653/1972; Vietor, 1884; Sweet, 1890; Honikman, 1964; Laver, 1978; Jenner, 2001; other terms include ‘voice quality setting’ and ‘basis of articulation’). The validity of the concept was first experimentally verified by Gick et al. (2004) using old x-ray data; the authors found that interspeech postures (ISPs) “assumed between speech utterances: (a) are language-specific; (b) function as active targets; (c) are active during speech, corresponding with the notion of ASs [articulatory settings], and (d) exert measurable influences on speech targets, most notably including effects on the properties of neutral vowels such as schwa” (p. 231). These findings have since been replicated across languages (Wilson et al., 2007; Wilson and Gick, 2014) and dialects (Wieling and Tiede, 2017), and Ramanarayanan et al. (2013) were able to show that ISPs also differ across speech styles (read vs. spontaneous speech) using real-time MRI.

It is conceivable that brass players might (a) use their native-language specific articulatory setting as default position during rests from playing and/or (b) develop a language- and brass playing-specific inter-playing position (IPP). A very limited comparison of only a single subject from each language group in this study in Heyne (2016) suggests that the latter indeed seems to be the case, and that the coronal place of articulation during both speech production and trombone playing heavily influences ISP and IPP. Note, however, that ISPs and IPPs are much harder to measure than vowels since either occur much less frequently, and the latter is even more so the case for IPPs due to the frequent occurrence of deep in-breaths during rests from playing, which require a very open vocal tract. Ultimately, it may not be necessary to measure AS/ISPs (and IPPs) separately, as suggested by an observation from Wieling and Tiede (2017) where they compare findings on ISPs across Dutch dialects to their earlier findings on tongue movements during word pronunciation (Wieling et al., 2016) within the same data set; they found that for both vowels and ISPs, one dialect group featured a more posterior tongue position than the other (measured using EMA), concluding that “articulatory setting differences may also be observed when analyzing a sizeable amount of variable speech data (i.e., not only focusing on a single segment)” (Wieling and Tiede, 2017, p. 392).

### Other Constraints on Tongue Shape During Brass Instrument Performance

It seems self-evident that brass playing imposes constraints upon vocal tract shape that differ substantially from speech production, not least the fact that the former generally requires a greater amount of airflow than the latter. The openness of the vocal tract was already touched upon above in relation to vowel tongue shapes, specifically neutral/central schwa which has long been viewed as effecting the least constriction in the vocal tract (Fant, 1960; Silverman, 2011; among many others). Early studies using MRI (e.g., Baer et al., 1991) have indeed shown that the vocal tract is heavily constricted in the oral cavity when producing high front vowels but the same also applies to the pharyngeal cavity when producing low back vowels. Either extreme would thus seem ill-suited for brass playing, providing a straightforward rationale for the midsagittal tongue shapes we observed across both groups. For the Tongan players, positioning the back of the tongue in a location similar to the ones used during the articulation of the back vowels /o/ and /u/ might provide the optimal solution given the aero-dynamical constraints of brass playing. Based on the assumption that the pharyngeal constriction for Tongan vowels would be at least somewhat comparable to the data for English by Baer et al. (1991), the vocal tract configurations of the Tongan vowels /i/ and /u/ might be too constrained in the oral cavity, while the low vowel /a/ might be too constrained in the pharyngeal cavity.

**FIGURE 11:**
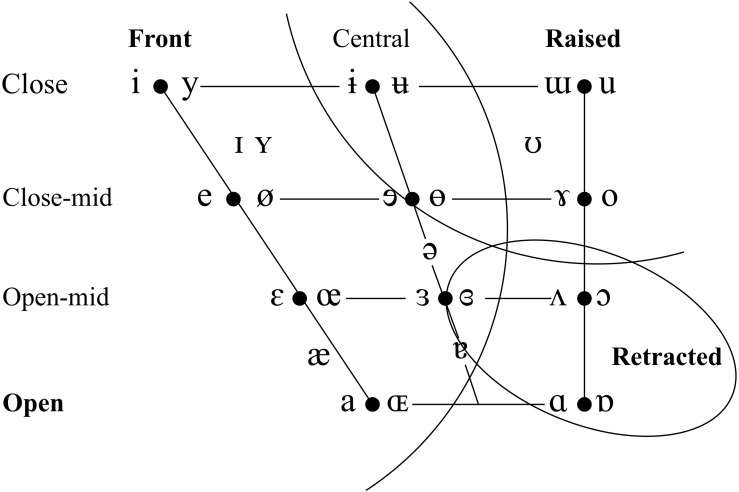
Revised vowel chart showing the division and overlap of articulatory regions. Reproduced with permission from Esling (2005, p. 23).

An alternative way of regarding the articulatory correlates of vowel tongue shape is suggested in Esling’s (2005) paper “There Are No Back Vowels: The Laryngeal Articulator Model.” Esling presents an attempt at re-conceptualizing the traditional vowel quadrilateral based on articulatory evidence on pharyngeal phonetics, adding the classifications “raised” and “retracted” to the traditional IPA chart (1996 version), as shown in Figure 11. Interestingly, the average midsagittal tongue shapes used by the musicians in our two language groups either straddle the boundaries of Esling’s re-categorization^7^ (NZE non-final /Ə/ and final schwa /Ə #/) or fall within the raised category (Tongan /o/ and /u/). Note that LOT (/ɒ/) in NZE is not a low vowel as shown on the IPA chart underlying Esling’s re-categorization, and is generally articulated somewhat closer (cf. Figure 1 in the introduction); our average tongue traces (Figure 7A) additionally suggest it is also somewhat fronted, definitely more so than the THOUGHT vowel (/o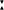/). By raising, Esling refers to “the positioning of the tongue when it is high (pulled upward and backward),” in contrast to retracted vowels, for which tongue position represents a “response to the sphinctering mechanism that closes the larynx” (14); the former action would have consequences for the pharyngeal cavity that would seem advantageous concerning airflow and some acoustical considerations affecting vocal tract resonances during brass playing (see below). A more recent conference paper (Moisik et al., 2019) provides some empirical support for the proposal that vowels pattern as front, raised and retracted in terms of larynx height in the form of MRI data collected from two subjects.

### Possible Acoustical Consequences of the Observed Language Differences

Throughout this paper we have discussed tongue shapes during vowel production and trombone playing from an articulatory perspective but it should be clear that we expect them to have acoustical consequences not only during speech production but also when playing the trombone. Basically, any changes to vocal tract shape will alter its acoustic impedance which will probably have an impact on instrument sound, even if the exact details of such a mechanism are of yet unknown. In a paper outlining considerations regarding vocal tract influence on different types of instruments, Wolfe et al. (2015) write that restricting the opening of the true vocal folds (or controlling their impedance) not only allows for “fine control of mouth pressure” but also affects potential vocal tract influence considerations by providing a “higher reflection coefficient for acoustic waves in the vocal tract” (p. 3). The result would be a reduced influence of subglottal resonances on upper vocal tract resonances (extending from the glottis to the lips, cf. citations listed in introduction) which interact with oscillations within the instrument (i.e., vocal tract influence), and which in turn would make it easier to adjust the vocal tract impedance peak falling within the frequency range of the trombone. That range cuts off around 700 Hz (for details see Campbell and Greated, 1987, p. 346–347) and given that vocal tract impedance peaks have a relationship of around 4/3 times the frequency of speech formants (depending on glottis openings, cf. Hanna et al., 2012), it would seem advantageous in terms of maximizing the potential for vocal tract influence, to assume a vowel tongue shape that produces formants below 900 Hz. In terms of F2, this suggests utilizing the back of the vowel space, while for F1 most vowels would fall within the range of the trombone. Unfortunately, empirical findings on glottal aperture during brass instrument performance are inconclusive, with observations from x-ray imaging (Carter, 1969; cf. Nichols et al., 1971), as well as real-time MRI (Iltis et al., 2017), suggesting that glottis opening is correlated closely with loudness (smaller opening during soft playing). However, other authors have reported that glottis aperture during playing may be “self-adjusting or involuntary” (Bailey, 1989, p. 105) or differ with proficiency level (professional players of all wind instruments had smaller glottal apertures than amateur and intermediate players in Mukai’s (1989) study, reported by Yoshikawa, 1998). Even though the latter finding would seem to fit well with Wolfe and colleagues’ consideration mentioned above, we are unable to draw any conclusions based on it given the variety of playing proficiencies included in our sample (within and across the two language groups).

While we were unable to perform acoustical analyses of the musical passages performed by all participants in this study due to audio quality, we conducted a limited comparison of recordings by two earlier participants of this study (S5 NZE and a semi-professional Japanese trombone player) who differed in their average tongue shape during trombone performance (Heyne and Derrick, 2015a). The Japanese player who used an /o/-like and thus more backed tongue position during playing (similar to the Tongan participants in this study) had a larger component of high frequencies in the produced sound spectrum compared to the NZE player who used a tongue shape resembling the group average for the NZE players in this paper; this result, however, should not be over-interpreted due to the small sample size and a possible confound in the different horizontal location of the narrowest oral constriction produced by the two subjects.

### Reconsidering the Role of Language Influence on Brass Instrument Performance

The previous paragraphs have outlined several constraints regarding tongue shape during brass instrument performance that we will now relate back to our initial discussion whereby motor memory from a player’s native language influences the tongue shape they employ when playing their instrument. Note that we regard language influence as secondary to any of these constraints, although there are certainly also interactions between language-related and -unrelated constraints, with the latter also affecting speech production, albeit probably to a lesser extent: Requirements of airflow favor the use of vocal tract configurations that avoid significant constrictions in the pharyngeal and/or oral cavities; high back vowels and non-low central vowels (optionally grouped as ‘raised’ in Esling’s (2005) ‘laryngeal articulator model’) seem to best satisfy these requirements. Considerations regarding the potential of vocal tract influence specific to the trombone suggest that a retracted (in the classical terminology) tongue position might be advantageous by situating the second vocal tract impedance peak (F2) below the cut-off frequency of the trombone (around 700 Hz). Furthermore, language influence via motor memory from a player’s native language might operate in a different, more direct manner by influencing the place of articulation used during trombone performance; our ultrasound videos include the relevant data but we have not been able to test this hypothesis yet.

### Confounds and Shortcomings of Our Study

Finally, we admit to the following shortcoming and confounds of our study: Our two language groups were quite heterogeneous not only in terms of participant age and instrumental experience but also in terms of playing proficiency; however, we placed greater emphasis on having sufficient participant numbers than keeping groups balanced as there were already a lot of other factors we were unable to control for such as how the individual players’ equipment (mouthpiece and instrument) might compare to performing on the ‘pBone.’ The group differences in tongue shape we found might be affected by individual vocal tract shape. It is plausible that the height and doming of our participants’ palates differed on the group level due to genetic factors (cf. Dediu et al., 2017, 2019; Dediu and Moisik, 2019) and this has been shown to impact speech production (see citations in introduction). All of our comparisons were based on large numbers of tokens collected at single time points during monophthong articulation (at 1/2 of vowel duration) and note productions (at 1/3 of note duration) and it has to be clear that this represents a simplification as neither activity is constant over time. Another confound is the use of a jaw brace tying ultrasound transducer position to jaw opening; while the system was shown to be relatively stable during speech production (Derrick et al., 2015), the same may not apply to brass instrument performance, and we did not carry out an assessment of motion variance in this context. However, no alternative ways of transducer stabilization compatible with trombone performance requirements were available at the time of data collection, and the use of any of the available systems for correcting for jaw position such as optical tracking systems (Mielke et al., 2005; Whalen et al., 2005; Miller and Finch, 2011; Noiray et al., 2015) would have exhausted the financial possibilities of a Ph.D. research project.

### Implications of Our Findings

Our findings show that two activities previously linked through their cognitive mechanisms, language and music, are also related more indirectly via motor memory resulting from a shared physiological system. Although both activities clearly represent forms of communication, the latter is inherently non-referential (if we disregard vocal music with lyrics), while the other is by definition referential or semiotic (but see Bowling et al., 2010; Curtis and Bharucha, 2010 for papers challenging this traditional distinction).

Our use of GAMMs for the analysis of midsagittal ultrasound tongue contours shows that SSANOVAs may be underestimating confidence intervals and hence overestimating statistical differences between tongue shapes produced in different contexts (cf. SSANOVA average curves of the same data set in Heyne, 2016). This would seem to be especially relevant for SSANOVA average curves calculated on the basis of small numbers of articulatory traces, unless phonetic context is tightly controlled for. GAMMs allow the inclusion of random smooths to model out the variance arising from independent variables and take the variance observed in different contexts into account when estimating the average curves and confidence intervals pertaining to a specific condition. In contrast, SSANOVAs do not afford these possibilities and it is unclear how one might correct for multiple comparisons if one would like to compare, e.g., articulations produced in more than two phonetic contexts.

## Conclusion

In this paper, we were able to present evidence for native language influence on brass instrument performance based on statistically robust differences determined using generalized additive mixed models (GAMMs) fit on large numbers of midsagittal ultrasound tongue contours collected during speech production and trombone playing. We argued that these differences can be related to the different vowels systems of the two languages groups observed in this study, New Zealand English and Tongan, but tongue shape during brass playing is more directly determined by constraints arising from airflow requirements and acoustical considerations. Our findings indicate that speech production, itself an acquired motor skill expressing a language’s underlying phonological system, can influence another skilled behavior, brass instrument performance, via motor memory of vocal tract gestures. More specifically, such vocal tract gestures would form the basis of local optimization processes to arrive at a suitable tongue shape for sustained note production, although further research is required to determine whether such behaviors occur across a larger population of players at various proficiency levels.

## Data Availability Statement

All datasets generated for this study are available on GitHub at: https://jalalal-tamimi.github.io/GAMM-Trombone-2019/.

## Ethics Statement

This study was carried out in accordance with the recommendations of the Ph.D. and Staff Low Risk Application Guidelines by the University of Canterbury Human Ethics Committee with written informed consent from all subjects. All subjects gave written informed consent in accordance with the Declaration of Helsinki. The protocol was approved by the University of Canterbury Human Ethics Committee (reference HEC 2014/02/LR-PS, February 2014).

## Author Contributions

MH and DD contributed to the conception and design of the ultrasound study and carried out all data collection. MH analyzed all ultrasound videos and organized all data into a combined database for statistical analysis. JA-T and DD performed the statistical analyses. MH prepared all visualizations and wrote the first draft of the manuscript. MH, DD, and JA-T wrote sections of the manuscript. All authors contributed to manuscript revision, read and approved the submitted version.

## Conflict of Interest

The authors declare that the research was conducted in the absence of any commercial or financial relationships that could be construed as a potential conflict of interest.

## Footnotes

1 https://jalalal-tamimi.github.io/GAMM-Trombone-2019/

2 It is possible to formally evaluate the addition of the random effect adjustments to the model. However, GAMM model estimation using Maximum Likelihood estimation (ML) is quite computationally demanding and we were unable to carry out full model comparisons for the largest model reported in this paper even when using a computing node with 36 cores and 1024 GB of memory!

3 https://jalalal-tamimi.github.io/GAMM-Trombone-2019/

4 https://jalalal-tamimi.github.io/GAMM-Trombone-2019/

5 https://jalalal-tamimi.github.io/GAMM-Trombone-2019/

6 https://jalalal-tamimi.github.io/GAMM-Trombone-2019/

7 Esling specifically comments that note that”[t]he intersection of the three lines dividing the three regions in [the figure] should perhaps fall exactly on the location of schwa to represent the focal point of movement away from neutral toward any of the three directions,” but that it was placed differently “to show the susceptibility of [ɐ] to becoming either front or retracted depending on the choice of articulator movement” (Esling, 2005, p. 23).

## Supplementary Material

FIGURE S1 Average smoothing splines for variance in tongue surface distance from the ultrasound virtual origin for NZE and Tongan note productions.

TABLE S1 Ultrasound recording parameters for all participants recorded for this study.
