# Supplementary figures and images for: Native Language Influence on Brass Instrument Performance: An Application of Generalized Additive Mixed Models (GAMMs) to Midsagittal Ultrasound Images of the Tongue
Source: Front Psychol. 2019 Nov 27;10:2597. doi: 10.3389/fpsyg.2019.02597 (PMC6890863; doi:10.3389/fpsyg.2019.02597)

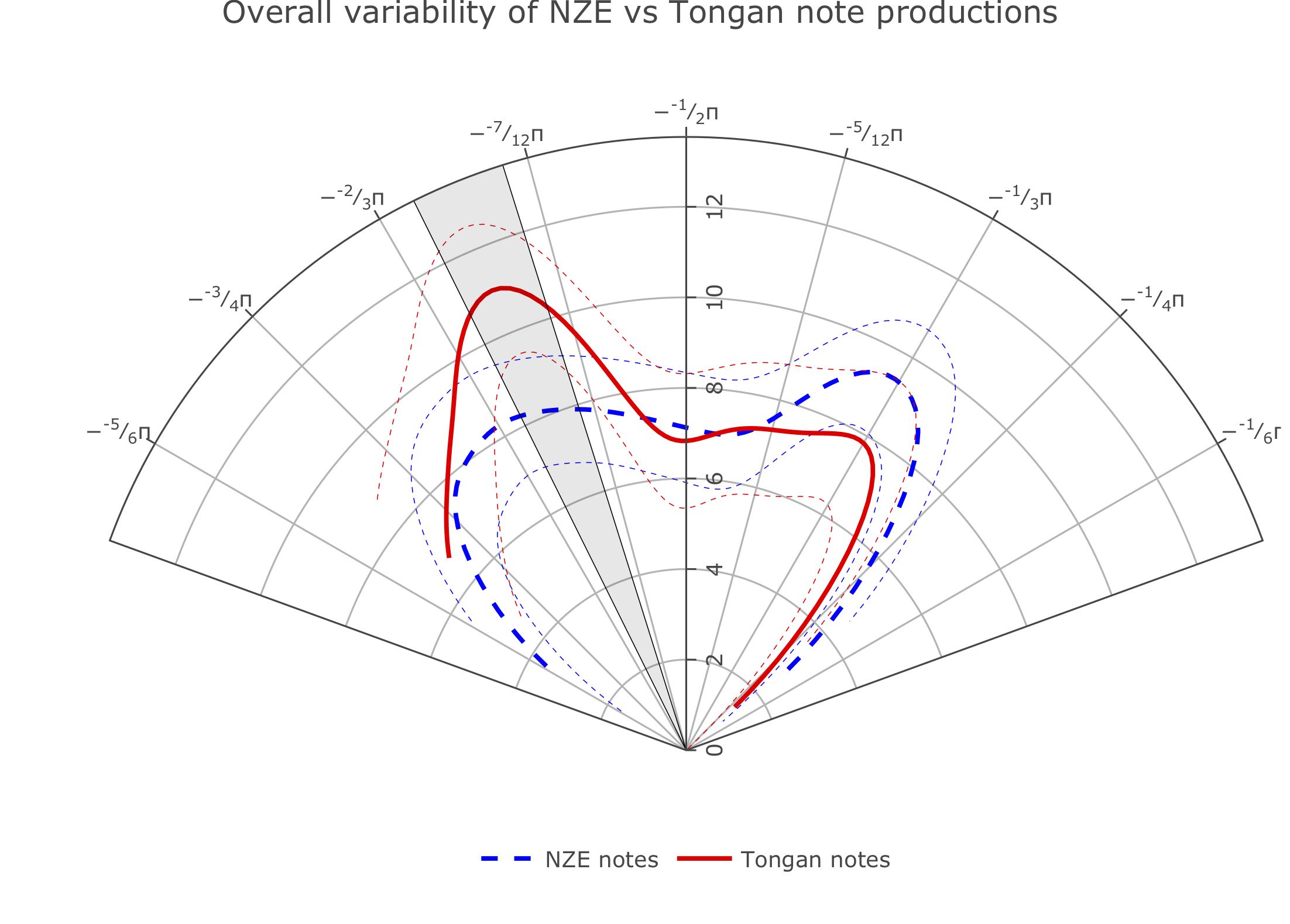

Supplement: FIGURE S1 — Average smoothing splines for variance in tongue surface distance from the ultrasound virtual origin for NZE and Tongan note productions. [file Image_1.JPEG]
